# Supplementary material for: Eco-Friendly Sanitization of Indoor Environments: Effectiveness of Thyme Essential Oil in Controlling Bioaerosol Levels and Disinfecting Surfaces
Source: BioTech (Basel). 2024 Apr 26;13(2):12. doi: 10.3390/biotech13020012 (PMC11130963; doi:10.3390/biotech13020012)
Supplement: Supplementary file 1 [file biotech-13-00012-s001.zip › biotech-2954770-supplementary.pdf]

Supplementary Materials: Eco-Friendly Sanitization of Indoor Environments: Effectiveness of Thyme Essential Oil in Controlling Bioaerosol Levels and Disinfecting Surfaces

Daniela Sateriale, Giuseppina Forgione, Giuseppa Anna De Cristofaro, Leonardo Continisio, Chiara Pagliuca, Roberta Colicchio, Paola Salvatore, Marina Paolucci and Caterina Pagliarulo

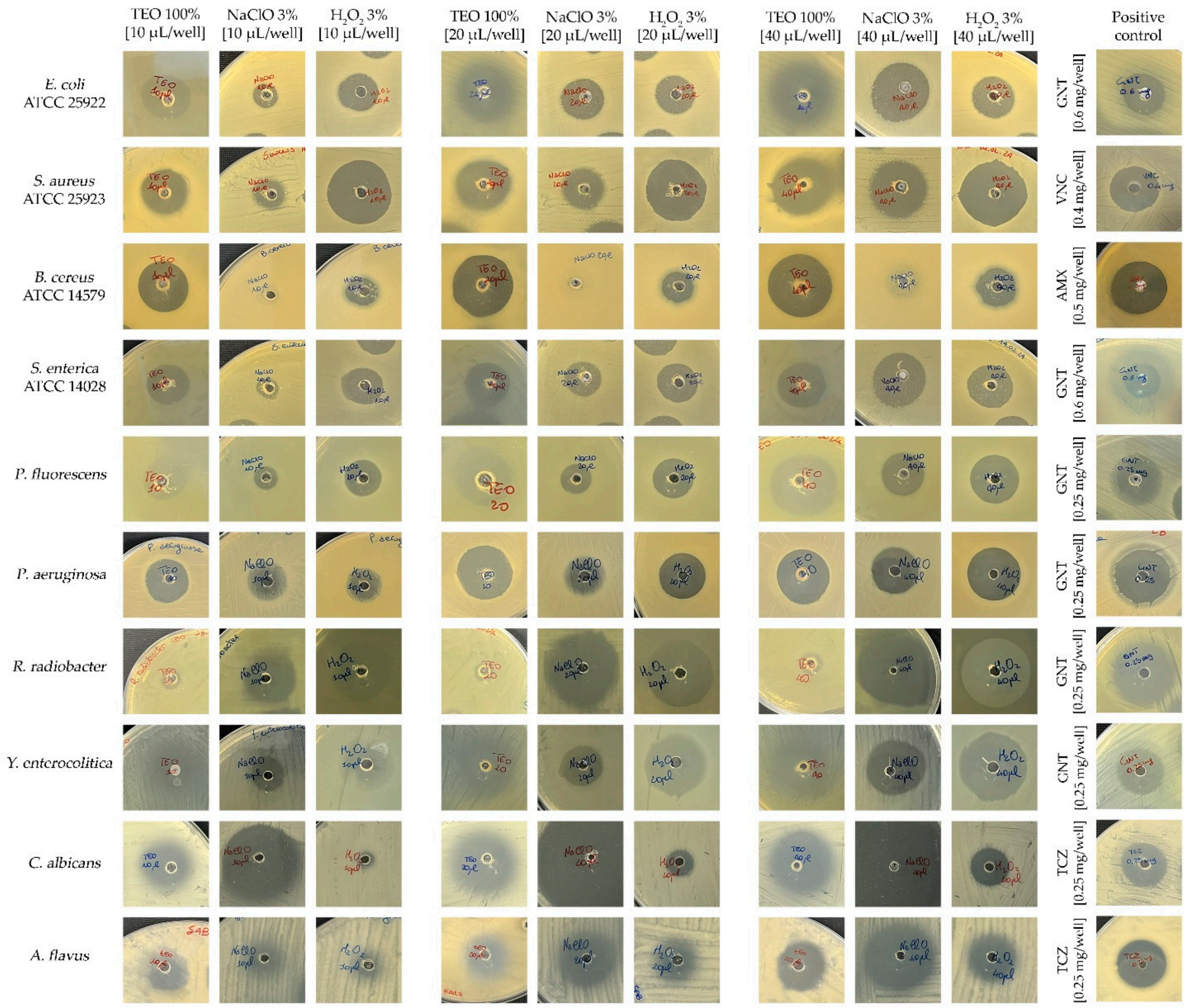

Figure S1. Images of *in vitro* antibacterial activity of thyme essential oil 100%, sodium hypochlorite solution 3%, hydrogen peroxide solution 3% and antimicrobials selected as positive controls, evaluated by the agar well diffusion method, against tested microorganisms. TEO, thyme essential oil; NaClO, sodium hypochlorite; H_2O_2, hydrogen peroxide; GNT, gentamicin; VNC, vancomycin; AMX, amoxicillin; TCZ, tioconazole.

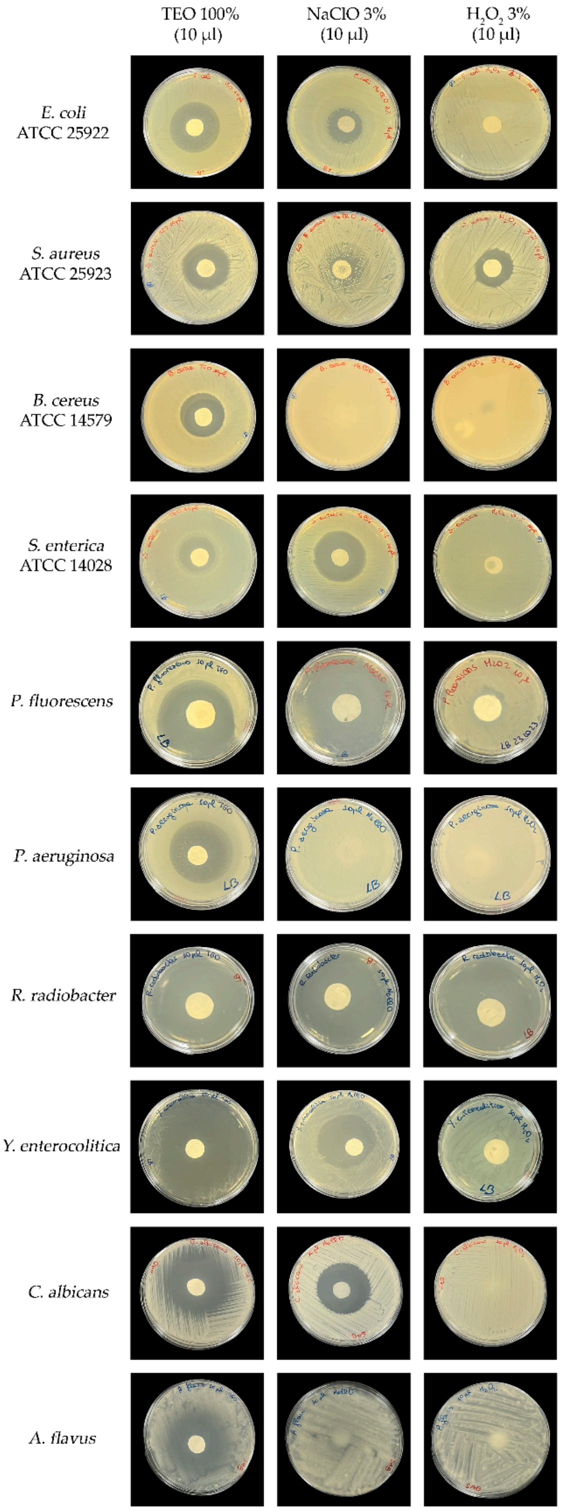

Figure S2. Images of *in vitro* antibacterial activity of thyme essential oil 100%, sodium hypochlorite solution 3% and hydrogen peroxide solution 3%, evaluated by disk volatilization method, against tested microorganisms. TEO, thyme essential oil; NaClO, sodium hypochlorite; H_2O_2, hydrogen peroxide.

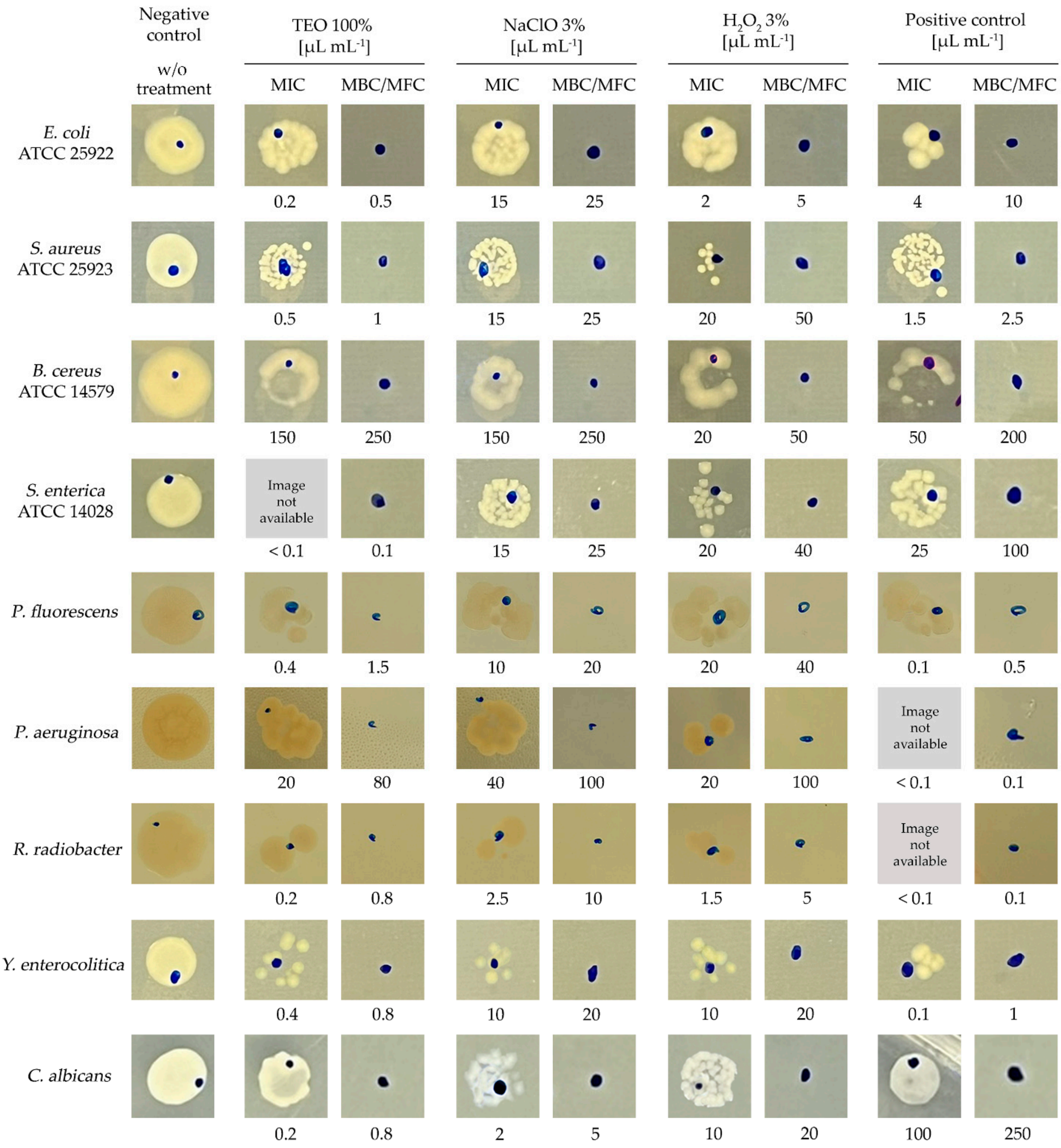

Figure S3. Images of quantitative evaluation of *in vitro* antibacterial activity of thyme essential oil 100%, sodium hypochlorite solution 3%, hydrogen peroxide solution 3% and antimicrobials selected as positive controls against tested microorganisms. Negative control, absence of antimicrobial agents; TEO, thyme essential oil; NaClO, sodium hypochlorite; H_2O_2, hydrogen peroxide; GNT, gentamicin; VNC, vancomycin; AMX, amoxicillin; TCZ, tioconazole.
